# Increased Isolation of Extended-Spectrum Beta-Lactamase-Producing Escherichia coli From Community-Onset Urinary Tract Infection Cases in Uttarakhand, India

**DOI:** 10.7759/cureus.13837

**Published:** 2021-03-11

**Authors:** Nitin Kumar, Kuhu Chatterjee, Sangeeta Deka, Ravi Shankar, Deepjyoti Kalita

**Affiliations:** 1 General Practice, All India Institute of Medical Sciences Rishikesh, Rishikesh, IND; 2 Microbiology, All India Institute of Medical Sciences Rishikesh, Rishikesh, IND; 3 Microbiology, Fakhruddin Ali Ahmed Medical College and Hospital, Barpeta, IND

**Keywords:** esbl, extended spectrum beta-lactamase, uropathogen, urinary tract infection, uti, community-acquired urinary tract infection, ca-uti, drug resistance, anti-microbial resistance

## Abstract

Background: Management of community-acquired urinary tract infection (CA-UTI) relies heavily on empirical antibiotic therapy. Knowledge of the proportion of drug-resistant isolates especially extended-spectrum beta-lactamase (ESBL)-producing *Escherichia coli* (*E. coli*), and various risk factors for acquisition are essential.

Method: Outpatient-treated CA-UTI cases were enrolled (continuously for three months), and microbiological analysis of urine sample was performed for significant bacterial growth followed by identification of conventional and matrix-assisted laser desorption/ionization-time of flight (MALDI-ToF) spectrometry method. Subsequent drug resistance and phenotypic ESBL detection were as per guidelines of the Clinical Laboratory Standard Institute (CLSI, USA). Univariate and multivariate analyses (logistic regression) of known and relevant risk factors of ESBL *E. coli* were performed as per standard statistical technique, using the SPSS computer package (IBM Corp., Armonk, NY).

Results: Two hundred and forty-one samples (of 694 samples) yielded significant growth. Sixty-one of 131 (46.6%) *E. coli *isolates were found to be ESBL producers. Non-beta-lactam antibiotic resistance in ESBL producers was high compared to non-ESBL producers (e.g., 88.5% vs 42.3% for quinolone resistance, 80.3% vs 34.3% for gentamicin resistance, etc.). Multivariate analysis (after univariate analysis detected probable factors of a likely ESBL model) indicated significant associations of ESBL-producing *E. coli* with advancing age (>55 years), prior hospitalization in last one year, use of antibiotics in previous six months, and presence of comorbid illness such as diabetes mellitus and chronic lung disease.

Conclusion: High proportion of our community-acquired uropathogens are ESBL-producing *E. coli* and likely resistant to important antimicrobial agents such as quinolones, gentamicin, etc. Factors like advancing age, prior hospitalization, and antibiotic use, as well as comorbidities such as diabetes and chronic lung disease, may be strongly associated with ESBL *E. coli* and should be remembered^ ^while administering or preparing guidelines for empiric management of CA-UTI subjects.

## Introduction

Urinary tract infections (UTIs) are among the most commonly acquired infections in the community setting and are often treated empirically with antibiotics. The increasing emergence of drug resistance in uropathogens largely attributed to the non-judicious use of antimicrobials poses a great therapeutic challenge in UTI treatment [1,2]. For a rational empirical treatment protocol, it is important to know the local pattern of the antimicrobial resistance. This study analyzed the pathogen profile and risk factors associated with extended-spectrum beta-lactamase-producing Escherichia coli (ESBL-E coli) agents in community-acquired uropathogens from UTI cases attending our newly established tertiary care teaching hospital at Rishikesh, Uttarakhand. It is expected that such early data from a new and developing hospital will help in constructing a baseline database to monitor trends in drug resistance and risk factor for the future - all future studies and preventive measures will be benefited from such a baseline database for the sake of comparison.

## Materials and methods

We undertook this study as a part of an Indian Council of Medical Research (ICMR)-sanctioned Short Term Studentship (STS) project (ICMR, Ref. no 2017-02671) against the name of the first author. A total of 865 urine samples were collected at our hospital (All India Institute of Medical Sciences Rishikesh) from new, consecutive outdoor-treated suspected community-onset urinary tract infection patients. All urine samples were taken as part of the clinical work-up, and these were included in this cross-sectional laboratory-based study. The study was carried out from mid-June to mid-August 2017. UTI was defined as “community-onset” when the infection occurred among non-hospitalized patients or less than 48 hours after hospitalization [3,4]. Urine specimens were obtained from all patients with a medical doctor or senior nurse; midstream urine was collected in a sterile container and processed in the laboratory within two hours of collection. The consent of the patient or their guardian was obtained before urine specimens were collected. The following exclusion criteria were used:

(i) contaminated urine culture (more than three microorganisms),

(ii) urinary tract symptoms but not positive urine culture, and

(iii) patients with healthcare-associated UTI (i.e., hospitalized for 48 hours during the last 30 days).

Relevant clinical data from the patient’s medical records were obtained and recorded. The variables studied were age, sex, prior hospitalization in the previous one year, antibiotic therapy in the preceding six months before the infection, prior catheterization, the incidence of UTI > three times, diabetes, nephrolithiasis, prior steroid intake, presence of chronic lung disease, menopause, and any benign enlargement of the prostate (BEP).

Microbiological analysis

**Figure 1:**
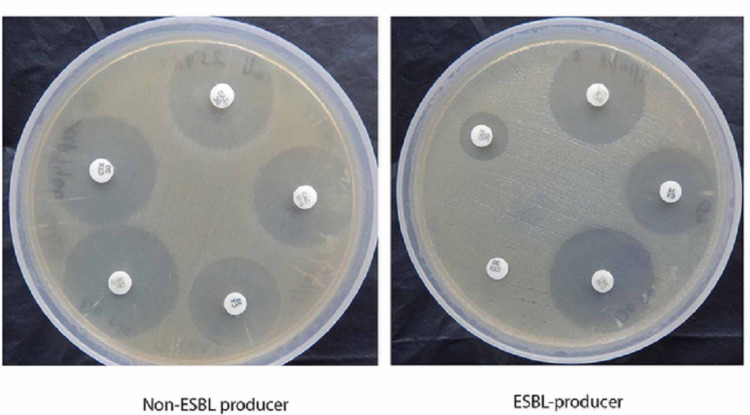
Phenotypic confirmatory test for ESBL (CLSI guidelines) ESBL, Extended-spectrum beta-lactamase; CLSI, Clinical Laboratory Standard Institute.

Using calibrated loops, urine specimens were inoculated on Cystine-Lactose-Electrolyte-Deficient (CLED) agar plates. After 18- to 24-hour incubation at 37℃, the number of colony-forming units (CFU) was counted, and urine samples giving 105 CFU/mL of urine were considered significant (as per Kass criteria) and selected for further processing. Double growth (two different colonies), each with significant growth individually, was considered for processing [5]. Subsequent identification of isolates was performed by both the conventional and Maldi-TOF systems (Bruker Corporation, Billerica, USA) [3]. Antibiotic susceptibility testing was carried out by conventional disk diffusion methods as per Clinical Laboratory Standard Institute (CLSI) 2018 guidelines. Phenotypic confirmation of ESBL production in *E. coli *isolates was performed by the method described in CLSI guidelines (Figure 1) [6].

Statistical analysis

ESBL-producing *E. coli *was further analyzed for potential risk factors of acquisition by univariate and multivariate analyses with the help of Statistical Package for Social Sciences software (SPSS 17.0, IBM Corp., Armonk, NY). Pearson Chi-square test (when all cells in 2 x 2 contingency table [for the association of factors risk factors with independent groups] is > 5) or Fisher’s exact test (if any of the association analysis 2 x 2 contingency table cells contain value less than 5) was applied to compare the categorical variables. A stepwise conditional logistic regression analysis was used to control the effects of confounding variables and identify the independent risk factors of infection. All risk factors with a p value of 0.1 (or less) at the bivariate level were included in the multivariate logistic model predicting ESBL-producing *E. coli*. All variables for which p was 0.05, in the multivariate analysis, were retained in the final model. Interactions between variables were not introduced into the models. Odds ratios (ORs) and their 95% confidential intervals (CIs) were calculated. All p values were two-tailed, and a p value of 0.05 was considered statistically significant.

Known risk factors for drug resistance such as age above 55 years, previous hospitalization in last one year, current catheter use, antibiotic use in last six months, previous UTI episode in last one year, presence of comorbid illnesses (chronic obstructive pulmonary disease [COPD], surgery for any indication, malignancy, etc.), presence of diabetes mellitus, onset of menopause, BEP, etc. were analyzed. 

## Results

Out of 865 selected cases (samples), 171 were excluded due to incomplete data (n = 134), resampling failure (n = 31), and visible contamination (n = 6). Of the remaining 694 samples, 241 (34.7%) yielded significant growth. Baseline features of these 241 (252 isolates, 11 samples had double isolation) cases are summarized in Table 1. A total of 252 isolates were identified, in which 249 were bacterial isolates, and the rest three being fungal (all Candida species) as tabulated in Table 2.

**Table 1:** Baseline profile of the 241 cases with positive growth

Characteristics	Total
Male:Female	49:192 (1:3.9)
Mean age (+SD)	39.2 (+18.79)
Double growth:Single growth	11:241

**Table 2:** Isolates profile

S. No.	Isolates	Total (n = 252)	Percentage
1	E. coli	131	52%
2	Klebsiella spp.	47	18.7%
3	Proteus spp.	2	0.8%
4	Enterobacter spp.	2	0.8%
6	Pseudomonas spp.	6	2.4%
7	Staphylococcus spp.	51	20.2%
9	Enterococcus spp.	10	4%
10	Candida spp.	3	1.2%

Drug resistance

ESBL phenotypic confirmatory testing (Figure 1) displayed 46.6% (61/131) *E. coli* isolates to be positive for ESBL production. All these strains (n = 61) were found to have reduced sensitivity/resistance for third-generation cephalosporin like ceftazidime (30 µg disk) in the initial disk-diffusion screening test.

ESBL-producing *E. coli* isolates were resistant to non-beta lactam antibiotics more than non-ESBL-producing *E. coli *isolates. Fluoroquinolone resistance rate was 88.5% and 42.3% (p < 0.0001), respectively, in these two groups. Some other important antibiotics are shown in Table 3.

**Table 3:** Antibiotic resistance among the ESBL and non-ESBL-producing E. coli isolates ESBL, Extended-spectrum beta-lactamase.

Antibiotics	ESBL-*E. coli* (n = 61)	Non-ESBL-*E. coli* (n = 70)	p value
Cotrimoxazole	53 (86.9%)	51 (72.9%)	0.7654
Nitrofurantoin	18 (29.5%)	16 (22.5%)	0.3864
Imipenem	2 (3.3%)	0	
Quinolones	54 (88.5%)	30 (42.3%)	<0.0001
Cefotaxim/ceftazidine/ceftriaxone	61 (100%)	7 (10%)	
Pipercillin-tazobactam	25 (41%)	6 (8.6%)	<0.0001
Amoxy-clav	61(100%)	16 (22.9%)	<0.0001
Amikacin	22 (36.1%)	8 (11.4%)	0.007
Gentamicin	49 (80.3%)	24 (34.3%)	<0.0001

Risk factor analysis

Table 4 depicts the results of univariate analysis risk factors for acquisition of ESBL-producing *E. coli*.

**Table 4:** Factors associated with ESBL-positive E. coli as uropathogen (univariate analysis with Chi-square or Fisher’s exact test) ESBL, Extended-spectrum beta-lactamase; UTI, urinary tract infection; BEP, benign enlargement of prostate gland.

Risk factors	ESBL positive	ESBL negative	p value
Sex, male	22	11	0.007
Age > 55 years	28	8	0.0001
Prior hospitalization last 1 year	26	6	0.0001
Antibiotic use in previous 6 months	43	14	0.0001
Fluoroquinolone	15	6	0.013
Cephalosporine	14	4	0.004
Other beta-lactams	14	4	0.004
Prior catheterization	11	6	0.108
Prior UTI > 3 times	14	9	0.115
Diabetes	25	6	0.0001
Nephrolithiasis	9	9	0.778
Prior steroid intake	8	6	0.243
Presence of chronic lung disease	15	7	0.017
Menopause	11	4	0.01
BEP	6	2	0.258 (Fisher’s exact test)
Other comorbidities	28	31	0.368

Male gender, age ≥ 55 years, history of hospitalization within one year, use of antibiotics within last six months, having diabetes mellitus, presence of chronic lung disease, and being in menopause were significant risk factors for ESBL-producing *E. coli* among community-onset UTI cases in univariate analysis (p < 0.05). Regarding antibiotic class, previous use of cephalosporin, fluoroquinolone, and other beta-lactams was associated with ESBL-producing *E. coli *(p = 0.004, p = 0.013, and p = 0.004, respectively).

Multivariate analysis for causation of ESBL-producing *E. coli*-linked UTI was modeled with factors yielding significant p values in Table 4 (i.e., male sex, age > 55 years, prior hospitalization in the previous one year, antibiotic use in previous six months [not taking individual antibiotic influence in the outcome], having diabetes mellitus, presence of chronic lung disease, and attainment of menopause). Out of these, only factors shown in Table 5 were found to statistically significant in multivariate analysis (logistic regression).

**Table 5:** Multivariate analysis (binomial logistic regression) of risk factors for UTI caused by ESBL-producing E. coli ESBL, Extended-spectrum beta-lactamase; UTI, urinary tract infection.

Factors	Odds ratio	95% Confidence interval	p value
Age above 55 years	6.41	1.27-32.3	0.024
Prior hospitalization in last 1 year	7.1	1.06-47.7	0.044
Antibiotic use in last 6 months	11.03	2.38-51.1	0.002
Diabetes	15.3	2.9-80.2	0.001

## Discussion

Isolation of ESBL-producing *E. coli* in community-onset UTI seems to be relatively common (e.g., in the current study, 61 out of 252 total isolates and about 52% of all *E. coli* isolates) with risk factors such as previous hospitalization, previous use of antibiotics, advancing age (>55 years), presence of comorbid illness like diabetes mellitus, etc.

Our study on community-onset UTI found significant growth in one-third of samples (34.7%, i.e., 241 out of 694 samples). Predominant isolates were *E. coli* (52.0%), *Klebsiella sp.* (18.7%), *Staphylococcus sp*. (20.2%), *Enterococcus sp.* (4%), other Gram-negative organisms, and three *Candida sp*. Among the *E. coli* isolates, 46.6% (61/131) isolates were ESBL producers, being more resistant to many non-beta lactam antibiotics (compared to non-ESBL-producing *E. coli*) like quinolone (88.5% vs. 42.3%), gentamicin (80.3% vs. 34.3%), etc. ESBL-producing *E. coli* showed more resistance to piperacillin-tazobactam combination than non-ESBL producers (41% vs. 8.6%). A large study (with 2,229 UTI samples) in 2017 found out 64.78% ESBLs, 15.97% carbapenem-resistant *Enterobacteriaceae*, and 42.7% multidrug-resistant (MDR) *Enterobacteriaceae *(MDRE), rates which were quite comparable to our findings [7]. *E. coli*, *Staphylococcus spp*., and *Pseudomonas spp.* emerged to be the most prevalent uropathogens in an Iranian study conducted in 2017 [8]. Earlier, Jena et al. found 38.62% MDR uropathogens of which 51.78% and 17.85% isolates were ESBLs and mixed resistance (e.g., MBL), respectively [9]. In their three-year-long study, they found that China, Vietnam, India, Thailand, and the Philippines had the maximum isolation ESBL-producing GNBs (gram-negative bacilli) and the highest rates of cephalosporin resistance. A high percentage of ESBL-producing *E. coli* strains were susceptible to amikacin and piperacillin-tazobactam. Very similar to our study, they found high resistance against the quinolones [10].

Statistical analysis (Chi-square/Fisher’s exact test) showed male gender, age > 55 years, prior hospitalization in last one year, previous antibiotic use in last six months (quinolone, cephalosporin, or other beta-lactams), presence of concurrent diabetes, chronic lung disease, and attaining menopause are to be a risk factor for acquisition of ESBL-producing *E. coli *(Table 4). Multivariate analysis (logistic regression) identified age above 55 years, prior hospitalization, and antibiotic use in recent six months, and diabetes is the significant risk factor for ESBL-producing *E. coli* (Table 5). One Thailand-based study on ESBL-producing uropathogenic *E. coli* found previous use of penicillin and cephalosporin, previous UTI, and earlier catheter use as an independent risk factor for ESBL producer acquisition [11]. A Taiwan-based study also found the susceptibility pattern of healthcare-associated UTI-causing *E. coli* to be similar to that of community-acquired UTI strains; however, a significant difference was noted with catheter-associated UTI-causing strains. ESBL production was higher in catheter-associated cases (30.0% vs. 3.5%) [12]. A recent Jordanian study found that the previous hospitalization can be an important independent risk factor of ESBL-producing *E. coli*-induced UTI. They also suspected previous antibiotics, renal abnormalities, etc. as other possible causes. High resistances to gentamicin, quinolone, amoxicillin-clavulanic acid combination, etc. were some other features similar to our current study [13]. One Korean team also found high resistance to quinolone and cotrimoxazole in ESBL-producing *E. coli* from UTI. But a majority of isolates were sensitive to aminoglycosides. Unlike our study, they found the risk factors for acquiring ESBL-producing *E. coli* mainly from previous UTI, cerebrovascular accident, and previous hospital infection [14]. Recently, a Turkey-based study reported 50.5% of UTI isolates to be ESBL-producing *E. coli*. Significant risk factors for ESBL-producing *E. coli* detection among community-acquired UTI cases were the use of antibiotics in the preceding six months, upper UTI, having multiple risk factors, etc. [4]. Another study on Korean female UTI patients found ESBL-producing *E. coli* in 17.7% of cases, with CTX-M 15 types being most common. Significant risk factors for ESBL-producing *E. coli *acquisition were recurrent vaginitis, UTI, and previous antibiotic use [15]. One recent Sri Lankan study found 46% ESBL-producing *E. coli* in CA-UTI cases belong to a few particular molecular types of ESBLs, particularly CTX-M, OXA, TEM, and SHV genes. In this study, about 41% of isolates were resistant to quinolone; however, isolates were largely sensitive to nitrofurantoin, aminoglycosides, fosfomycin, carbapenems, etc. [16]. An African study found ESBL-producing uropathogen in 23% of isolates, out of which more than 75% were *E. coli*. Previous antibiotic use and recurrent UTI were identified as significant risk factors [17]. Our current study is carried out in a relatively less exposed population to antibiotics, being in a newly established institute from an underdeveloped hilly province of India. However, to have more comprehensive knowledge, another study with molecular typing of ESBLs can be done (based on current data).

## Conclusions

A prevalence rate of 46.6% ESBL producer as community-acquired uropathogen is a high rate of drug resistance. Identified risk factors such as previous antibiotics, hospital admission, advancing age, and co-morbidity like diabetes and chronic lung diseases will be useful inputs for a future effective antibiotic policy for empirical management of outdoor UTI cases (in our population). High quinolone resistance and low-to-medium resistance to aminoglycosides can be other essential issues arising from this study.

## Appendices

Abbreviations used

UTI = Urinary tract infection.

COPD = Chronic obstructive pulmonary disease.

ESBL = Extended-spectrum beta-lactamase.

MDR = Multi-drug resistance.

CA-UTI = Catheter-associated UTI.

CTX-M, OXA, TEM, and SHV = Molecular types of extended-spectrum beta-lactamase enzyme released by bacteria (ESBL properties).

## References

[1] Sharma S, Bhat GK, Shenoy S (2007). Virulence factors and drug resistance in Escherichia coli isolated from extraintestinal infections. Indian J Med Microbiol.

[2] Linhares I, Raposo T, Rodrigues A, Almeida A (2013). Frequency and antimicrobial resistance patterns of bacteria implicated in community urinary tract infections: a ten-year surveillance study (2000-2009). BMC Infect Dis.

[3] Duguid J, Collee J, Fraser A. Duguid JP, Collee JG, Fraser AG (1989). Laboratory strategy in the diagnosis of infective syndromes. Mackie & McCartney Practical Medical Microbiology (13th ed.).

[4] Tüzün T, Kutlu SS, Kutlu M, Kaleli İ (2019). Risk factors for community-onset urinary tract infections caused by extended-spectrum β-lactamase-producing Escherichia coli. Turk J Med Sci.

[5] Livermore DM, Canton R, Gniadkowski M (2007). CTX-M: changing the face of ESBLs in Europe. J Antimicrob Chemother.

[6] Patel JB (2017). Performance Standards for Antimicrobial Susceptibility Testing: Twenty-Third Informational Supplement (CLSI document M100-S23). Villanova, Pa, USA: Clinical and Laboratory Standards Institute.

[7] Banerjee S, Sengupta M, Sarker TK (2017). Fosfomycin susceptibility among multidrug-resistant, extended-spectrum beta-lactamase-producing, carbapenem-resistant uropathogens. Indian J Urol.

[8] Mihankhah A, Khoshbakht R, Raeisi M, Raeisi V (2017). Prevalence and antibiotic resistance pattern of bacteria isolated from urinary tract infections in Northern Iran. J Res Med Sci.

[9] Jena J, Debata NK, Subudhi E (2013). Prevalence of extended-spectrum-beta-lactamase and metallo-beta-lactamase producing multidrug resistance gram-negative bacteria from urinary isolates. Indian J Med Microbiol.

[10] Jean S S, Coombs G, Ling T (2016). Epidemiology and antimicrobial susceptibility profiles of pathogens causing urinary tract infections in the Asia-Pacific region: results from the study for monitoring antimicrobial resistance trends (SMART), 2010-2013. Int J Antimicrob Agents.

[11] Savatmorigkorngul S, Poowarattanawiwit P, Sawanyawisuth K, Sittichanbuncha Y (2016). Factors associated with extended-spectrum Β-lactamase producing Escherichia coli in community-acquired urinary tract infection at hospital emergency department. Southeast Asian J Trop Med Public Health.

[12] Huang LF, Lo YC, Su LH, Chang CL (2014). Antimicrobial susceptibility patterns among Escherichia coli urinary isolates from community-onset healthcare-associated urinary tract infection. J Formos Med Assoc.

[13] Albaramki JH, Abdelghani T, Dalaeen A, Ahmad FK, Alassaf A, Odeh R, Akl K (2019). Urinary tract infection caused by extended-spectrum β-lactamase-producing bacteria: risk factors and antibiotic resistance. Pediatr Int.

[14] Lee H, Han SB, Kim JH, Kang S, Durey A (2018). Risk factors of urinary tract infection caused by extended-spectrum β-lactamase-producing Escherichia coli in emergency department. Am J Emerg Med.

[15] Kim YA, Lee K, Chung JE (2018). Risk factors and molecular features of sequence type (ST) 131 extended-spectrum-β-lactamase-producing Escherichia coli in community-onset female genital tract infections. BMC Infect Dis.

[16] Priyadharshana U, Piyasiri LB, Wijesinghe C (2019). Prevalence, antibiotic sensitivity pattern and genetic analysis of extended spectrum beta lactamase producing Escherichia coli and Klebsiella spp among patients with community-acquired urinary tract infection in Galle district, Sri Lanka. Ceylon Med J.

[17] Abayneh M, Tesfaw G, Abdissa A (2018). Isolation of extended-spectrum β-lactamase-(ESBL-) producing Escherichia coli and Klebsiella pneumoniae from patients with community-onset urinary tract infections in Jimma University Specialized Hospital, Southwest Ethiopia. Can J Infect Dis Med Microbiol.

